# Metanephric stromal tumor as a rare differential diagnosis of a renal mass in children – a case report

**DOI:** 10.1007/s00247-025-06166-w

**Published:** 2025-01-23

**Authors:** Ronja Riese, Simon Veldhoen, Corona Metz, Carolin Scale

**Affiliations:** Pediatric Radiology, Kinderradiologie, Charité - Universitätsmedizin Berlin, corporate member of Freie Universität Berlin and Humboldt-Universität Zu Berlin, Augustenburger Platz 1, Berlin, 13353 Germany

**Keywords:** Childhood kidney tumors, Computed tomography, Kidney, Magnetic resonance imaging, Metanephric stromal tumor, Nephroblastoma, Pediatric renal tumors, Ultrasound

## Abstract

This report presents the case of a benign metanephric stromal tumor that occurred in the kidney of a 5-year-old boy and in which differentiation from a nephroblastoma was successful. Radiological assessment played the decisive role in this case, as the low initial volume, a high apparent diffusion coefficient, and lack of rapid tumor growth were considered atypical for a nephroblastoma. The boy underwent successful kidney-preserving resection without neoadjuvant chemotherapy, and intraoperative contrast-enhanced ultrasound examination helped to accurately define the tumor margins. The case is reported to provide information on the radiological picture of pediatric metanephric stromal tumor.

## Introduction

Metanephric stromal tumor is a rare tumor that can arise in the kidney and mainly occurs in children. The entity was first described in 2000 by Argani et al., but to date there is little information about its incidence in the literature [1]. The median age at diagnosis is 2 years [2].

Knowledge of this rare tumor entity is particularly relevant as a benign differential diagnosis to the much more common and malignant nephroblastoma (syn. Wilms tumor), which accounts for around 90% of all childhood kidney tumors [3]. Malignant transformation has not been described for metanephric stromal tumor; however, one case has been described in which the same tumor entity recurred in the testis after successful resection of a metanephric stromal tumor from the kidney [4]. If the metanephric stromal tumor is recognized as such, affected patients can be spared both preoperative chemotherapy and total nephrectomy of the affected side. We report a case in which early diagnosis of a metanephric stromal tumor was successful.

## Case report

**Fig. 1:**
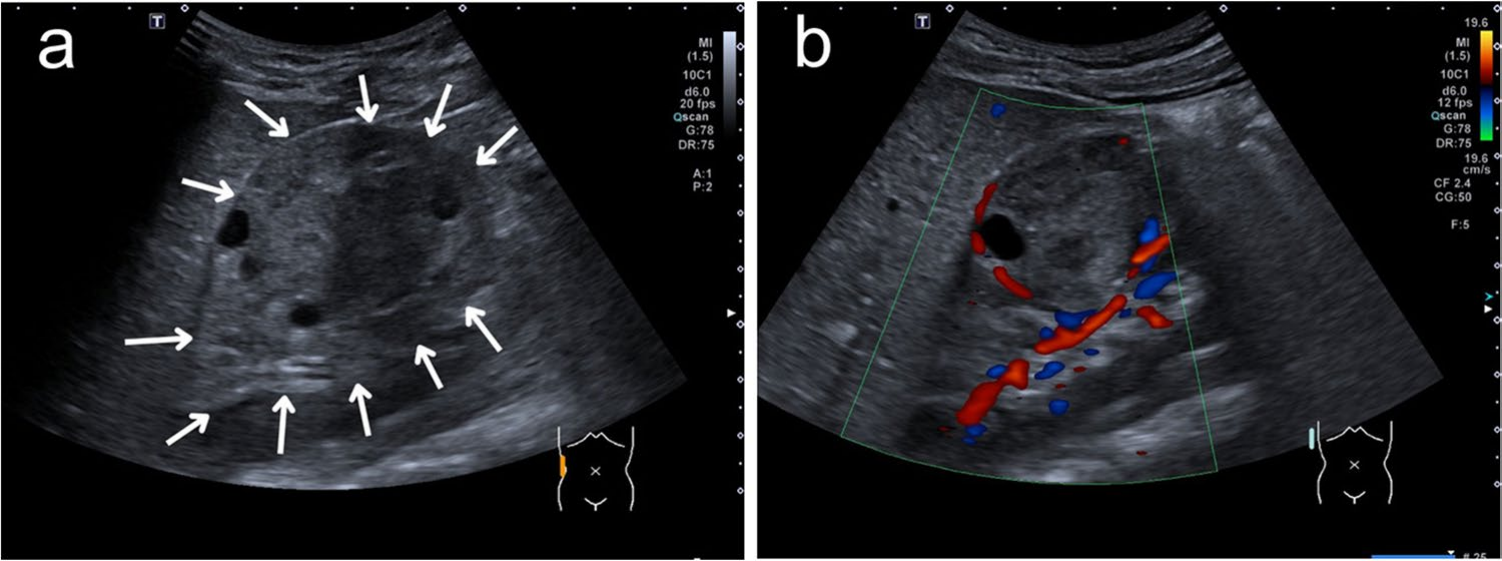
Longitudinal ultrasound image of the right kidney of a 5-year-old boy with a metanephric stromal tumor sharply demarcated from the renal parenchyma (*arrows* in **a**) and evidence of multiple internal cysts. Significantly reduced perfusion in the color Doppler image (**b**)

**Fig. 2:**
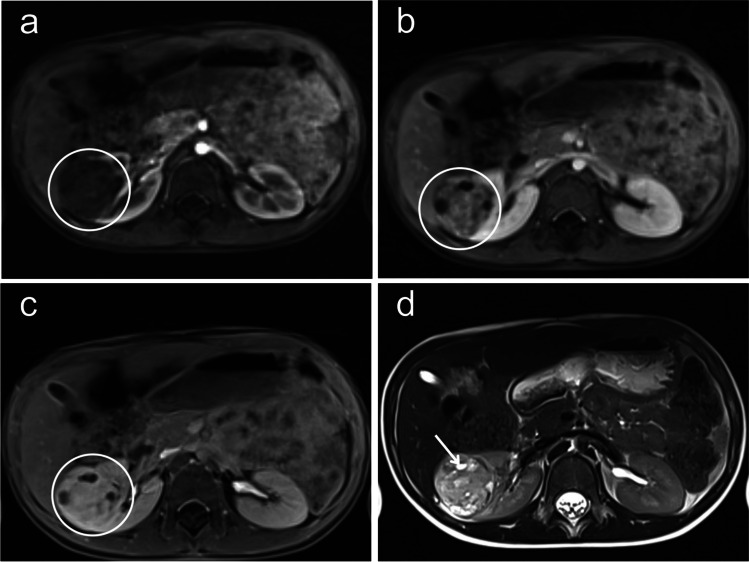
The contrast agent dynamics of the magnetic resonance images of the metanephric stromal tumor of a 5-year-old-boy shows relevant enhancement in the urographic phase only. **a** Dynamic axial T1-weighted volumetric interpolated breath-hold examination golden-angle radial sparse parallel (VIBE GRASP) magnetic resonance image with fat saturation shows delayed contrast agent uptake of the right-sided metanephric stromal tumor in the arterial phase compared to the renal parenchyma (*circle*). **b** The dynamic contrasted axial T1-weighted VIBE-GRASP magnetic resonance image shows the right kidney including the metanephric stromal tumor in the nephrographic phase with moderate enhancement. **c** The dynamic contrasted axial T1-weighted VIBE GRASP magnetic resonance image shows the right kidney including the metanephric stromal tumor in the urographic phase with enhancement comparable of the renal parenchyma. **d** Cystic (*arrow*) and solid parts are well demarcated in the axial T2-weighted half Fourier-acquired single shot turbo spin echo (HASTE) sequence of the magnetic resonance imaging

**Fig. 3:**
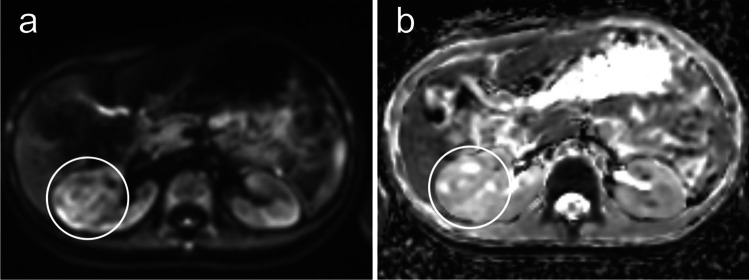
Axial magnetic resonance imaging of a 5-year-old boy. Absence of diffusion restriction of the metanephric stromal tumor (*circles* in **a** and **b**) in diffusion-weighted imaging, no apparent diffusion coefficient value reduction compared to the healthy kidney

**Fig. 4:**
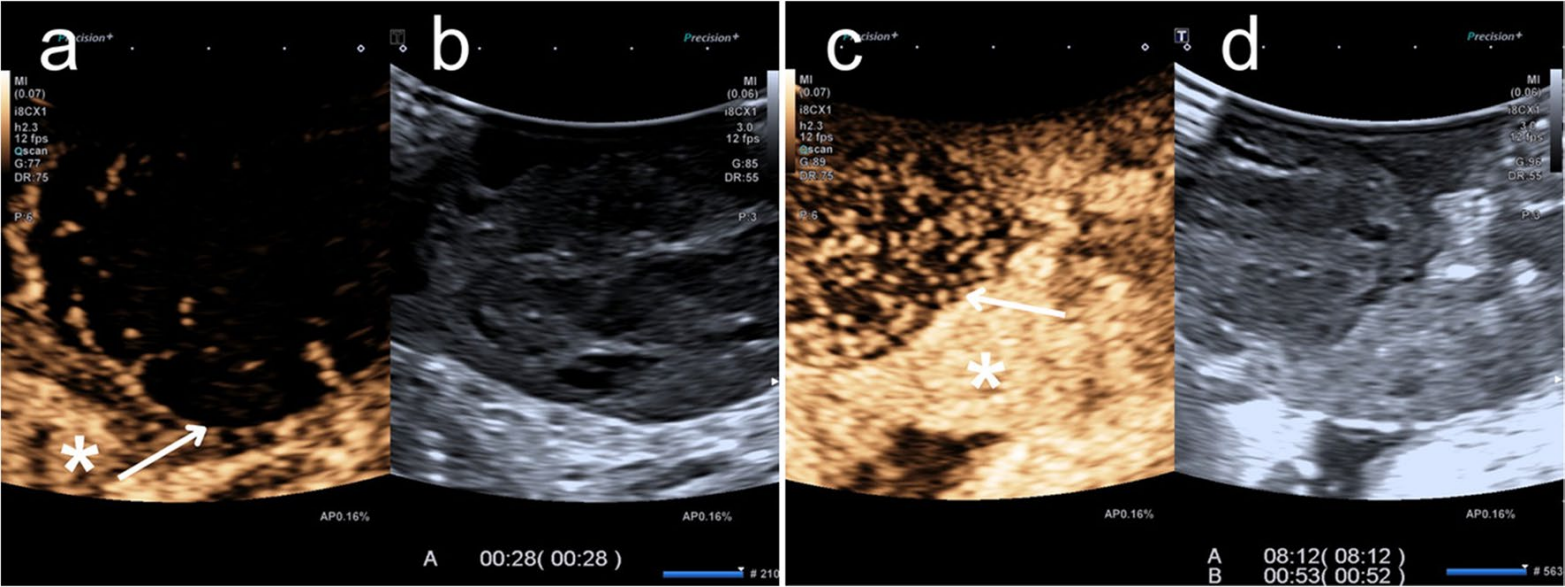
Intraoperative oblique contrast-enhanced ultrasound examination of the right kidney of a 5-year-old boy with reduced contrast agent uptake of the tumor tissue (*arrows*) compared to the healthy renal parenchyma (*asterisks*) in both the early (**a** and **b**) and late (**c** and **d**) contrast phase. **a**,** c** Contrast-enhanced sonographies. **b**,** d** Grayscale sonographies

A 5-year-old boy was referred from his pediatrician to the pediatric radiology department for sonography due to recurrent abdominal pain and suspected food intolerance. A sonogram of the abdomen was performed, which showed an inhomogeneous and, compared to the renal parenchyma, hypovascularized mass with a diameter of up to 5.3 cm and multiple internal cysts in the middle third of the right kidney (Fig. 1). Due to the strong suspicion of a nephroblastoma, a contrast-enhanced magnetic resonance imaging (MRI) was performed the next day. It revealed a sharply defined, questionably encapsulated mass measuring approx. 4 × 4 × 4.8 cm (40 ml) with an inhomogeneous, predominantly T2W intermediate signal and numerous cystic internal components (Fig. 2) without contact to the renal pelvis. The contrast agent dynamics showed a clearly delayed uptake compared to the renal parenchyma with strong enhancement in the urographic phase only (Fig. 2a-c). In the diffusion-weighted imaging (DWI), the mass showed a slightly increased signal and intermediate apparent diffusion coefficient (ADC) values (mean value approx. 1.0 × 10^–3^ mm^2^/s) (Fig. 3). There was no pathological enlargement of lymph nodes. The subsequent computed tomography (CT, computed tomography dose index 0.85 mGy) of the thorax ruled out pulmonary metastases 2 days after the MRI. An MRI of the head due to recurrent headaches also revealed no evidence of metastases. The low tumor volume and the lack of diffusion restriction in the DWI cast doubt on the primary imaging diagnosis of a nephroblastoma, where one would typically expect a hypointense signal in comparison to the kidney in T1 and an iso- or hyperintense signal in T2 with restricted diffusion on DWI in non-cystic areas of the tumor [7]. In addition to a nephroblastoma, a clear cell sarcoma and a rhabdoid tumor were also considered differential diagnoses. During the diagnostic work-up and until the external assessments of the responsible study center and reference radiology were received, continuous sonographic follow-up examinations were performed, which showed no progression in the size of the mass – which is unusual for nephroblastoma, which typically progresses rapidly. The tumor board decided to perform a fine needle biopsy 14 days after initial presentation. The pathology department initially considered an inflammatory myofibroblastic tumor but finally, 15 weeks and a second fine needle biopsy later, a metanephric stromal tumor with *BRAFV600E* mutation was diagnosed after multiple examinations in the pathology department and consultation of the reference pathology. Sixteen weeks after the initial presentation of the patient, the tumor was successfully resected in total with renal preservation with help of intraoperative visualization of tumor margins by contrast-enhanced ultrasonography (CEUS) (Fig. 4).

## Discussion

Only a few cases of metanephric stromal tumors have been described in the literature, which have mainly been diagnosed using CT as a preoperative diagnostic procedure followed by subsequent nephrectomy (see [5] for example). The starting point for diagnosis is usually – as in the case reported here – the ultrasound (US) examination, in which metanephric stromal tumor presents heterogeneously with solid and cystic parts, which can be isoechogenic, hypoechogenic, or hyperechogenic compared to the renal parenchyma [5]. On CT, a metanephric stromal tumor characteristically presents as a well-defined hypodense lesion, which shows varying proportions of cystic and solid structures. Strong contrast enhancement occurs in the urographic phase (see also [3]). Focal calcifications are also described, which may be helpful in the differentiation from nephroblastoma, which only rarely calcifies [2, 6]. In pediatric radiology, however, abdominal cross-sectional imaging is primarily performed using MRI for reasons of radiation protection. The characteristics of MRI diagnosis of pediatric metanephric stromal tumors have been little discussed in the literature to date. Kacar et al. describe a pediatric case in which the tumor appeared T1W hypointense and with a T2W hyperintense central cystic portion on MRI [6]. This description is similar to the case presented here. In contrast, Fan et al. describe the case of an adult patient whose lesion showed a T1W isointense signal and a T2W hypointense signal on MRI [5]. However, in view of the few case descriptions, it cannot be reliably postulated that the presentation varies according to age.

At present, it does not appear possible to make a reliable diagnosis of metanephric stromal tumor using radiological methods. However, radiology can play a decisive role in determining whether a tumor biopsy should be performed for further diagnosis, especially since nephroblastoma is the much more common diagnosis and does not require a biopsy before treatment. Jackson et al. have developed criteria to facilitate decision-making for or against performing a biopsy [8]. A biopsy is recommended if the tumor is of uncertain renal origin, a patient age >10 years, a tumor volume <200 ml at an age of 7–10 years or if it shows an atypical metastatic pattern [8]. In the presented case, the documented constancy in size of the tumor over several weeks cast doubt on the suspected diagnosis of nephroblastoma, which typically progresses rapidly. In addition to the criteria compiled by Jackson et al., the simultaneous presence of a constant lesion size, the absence of diffusion restriction, and the presence of multiple cysts were added by this case report as a combination that can be used to decide for a biopsy in the tumor board.

In summary, the diagnosis of rare pediatric renal tumors is known to be difficult based on radiological imaging alone. The low incidence of metanephric stromal tumors precludes any diagnostic routine from the outset, so that it seems advisable to make full use of the diagnostic spectrum, especially MRI.

If a renal mass is identified that cumulatively shows a small initial tumor volume, no size progression, no significant diffusion restriction, and multiple internal cysts, a tumor biopsy should be considered in order to rule out differential diagnoses of nephroblastoma. This can be of considerable consequence for patients, as nephroblastomas are usually treated without prior histologic confirmation by means of neoadjuvant chemotherapy and nephrectomy. Due to the characteristic hypovascularization of metanephric stromal tumors, CEUS is suitable for providing the surgeon with valuable information for the intraoperative determination of tumor margins in the context of kidney-preserving surgery and complete resection.

Consequently, the correct diagnosis can spare patients unnecessary chemotherapy and total nephrectomy.

## Data Availability

No datasets were generated or analysed during the current study.
